# RESTORE: Once-nightly oxybate dosing preference and nocturnal experience with twice-nightly oxybates

**DOI:** 10.1016/j.sleepx.2024.100122

**Published:** 2024-08-15

**Authors:** Asim Roy, Thomas Stern, John Harsh, J. Douglas Hudson, Akinyemi O. Ajayi, Bruce C. Corser, Emmanuel Mignot, Adrian Santamaria, Anne Marie Morse, Brian Abaluck, Sally Ibrahim, Paula K. Schweitzer, Katie Lancaster, Jordan Dubow, Jennifer Gudeman

**Affiliations:** a Ohio Sleep Medicine Institute, Dublin, OH, USA; b Advanced Respiratory and Sleep Medicine, PLLC, Huntersville, NC, USA; c Colorado Sleep Institute, Boulder, CO, USA; d FutureSearch Trials of Neurology, Austin, TX, USA; e Florida Pediatric Research Institute, Winter Park, FL, USA; f Sleep Management Institute, Cincinnati, OH, USA; g Center for Narcolepsy, Stanford University, Palo Alto, CA, USA; h Northwest Houston Neurology & Comprehensive Sleep Medicine Center, Houston, TX, USA; i Geisinger Commonwealth School of Medicine, Geisinger Medical Center, Janet Weis Children's Hospital, Danville, PA, USA; j Avadel Pharmaceuticals, Chesterfield, MO, USA; k University Hospitals Cleveland Medical Center, Cleveland, OH, USA; l Sleep Medicine & Research Center, St. Luke's Hospital, Chesterfield, MO, USA; m Patient Author, USA

**Keywords:** Narcolepsy, Sodium oxybate, Twice-nightly, Once-nightly, Adherence, Nocturnal adverse events

## Abstract

**Objective/Background:**

Preference for extended-release, once-nightly sodium oxybate (ON-SXB, FT218) vs twice-nightly immediate-release (IR) oxybate was assessed in participants switching from IR oxybate to ON-SXB in an open-label/switch study, RESTORE (NCT04451668).

**Patients/Methods:**

Participants aged ≥16 years with narcolepsy who completed the phase 3 REST-ON trial, were oxybate-naive, or were on a stable IR oxybate dose (≥1 month) were eligible for RESTORE. For participants who switched from twice-nightly dosing to ON-SXB, initial doses were closest or equivalent to their previous nightly IR oxybate dose. These participants completed a questionnaire at baseline about nocturnal adverse events associated with the middle-of-the-night IR oxybate dose in the preceding 3 months, a preference questionnaire after 3 months of stable-dose ON-SXB, and an end-of-study (EOS) questionnaire.

**Results:**

There were 130 switch participants; 92/98 (93.9 %) who completed the preference questionnaire preferred ON-SXB. At baseline, 69.2 % reported missing their second IR oxybate dose at least once; in these cases, 80 % felt worse the next day. Approximately 39 % reported taking a second nightly IR oxybate dose >4 h after the first dose, 51 % of whom felt somewhat to extremely groggy/unsteady the next morning; 120 participants (92 %) reported getting out of bed after their second oxybate dose. Of those, 9 (8 %) reported falls and 5 (4 %) reported injuries. Of the switch participants who completed the EOS questionnaire, 91.2 % felt better able to follow the recommended ON-SXB dosing schedule.

**Conclusions:**

The second nightly IR oxybate dose presents significant treatment burdens and adherence concerns. Participants overwhelmingly preferred the once-nightly dosing regimen of ON-SXB.

## Highlights

• Once-nightly sodium oxybate (ON-SXB) requires just 1 bedtime dose.
• Prior to ON-SXB approval, only twice-nightly oxybates were available.
• 80 % of twice-nightly switch patients who missed the 2nd dose felt worse the next day.
• 94 % of participants who switched from twice-nightly oxybate preferred ON-SXB.
• 93 % of participants would recommend ON-SXB to family or friends with narcolepsy.

## 1 Introduction

Narcolepsy is a chronic sleep disorder that usually requires lifelong disease management [1]. Sodium oxybate (SXB) is a recommended treatment for narcolepsy symptoms, including excessive daytime sleepiness (EDS) and cataplexy [2,3]. It is also effective for disrupted nighttime sleep (DNS) [3]. Twice-nightly immediate-release (IR) SXB has been an effective treatment for patients with narcolepsy for over 20 years [[4], [5], [6]]. SXB has been transformative for many patients, controlling symptoms better than previously used therapies (ie, antidepressants, stimulants) [7].

There are two twice-nightly IR oxybate formulations approved for the treatment of narcolepsy (SXB [composed only of sodium oxybate] and calcium/magnesium/potassium/sodium oxybates [mixed-salt oxybates]) [8,9]. The first nightly dose of IR oxybate is taken at bedtime, and the second 2.5–4 h later, requiring individuals to chronically awaken to take the second dose in the middle of the night [8,9]. Although oxybates are effective in treating narcolepsy symptoms [4,10,11], the dosing regimen of IR oxybate formulations can be burdensome for patients [12]. For example, oxybate prescribing is strictly regulated by a Risk Evaluation and Mitigation Strategy program [8,9], and patients may be afraid of potentially losing access, making them less likely to be forthcoming about difficulties they are experiencing with oxybate use. Anonymous surveys about the patient experience [12], as well as an analysis of the US Food and Drug Administration (FDA) Adverse Event Reporting System (FAERS) [13], have reported issues with missing the second dose altogether, taking it >4 h after the first dose, next-day consequences, and adverse events associated with dosing <2.5 h after the first dose [14].

Once-nightly, extended-release SXB (ON-SXB; FT218; LUMRYZ™, Avadel Pharmaceuticals, Chesterfield, MO) has shown efficacy in treating narcolepsy symptoms (ie, EDS, Clinical Global Impression of Improvement, cataplexy, and DNS) in the phase 3, placebo-controlled REST-ON clinical trial, with a safety and tolerability profile consistent with that of IR SXB [15,16]. ON-SXB is FDA approved for the treatment of EDS or cataplexy in patients with narcolepsy [17]. The single nightly dose of ON-SXB provides therapeutic coverage for the entire nocturnal sleep period, thus eliminating the need for a middle-of-the-night dose, and has been recognized as a major contribution to patient care [18]. As part of the open-label RESTORE study (NCT04451668), participants who switched from IR oxybate to ON-SXB were surveyed regarding their preference for a once-nightly vs twice-nightly dosing regimen and their previous experience with the second nightly IR oxybate dose.

## 2 Materials and Methods

### 2.1 Study design

RESTORE was a multicenter, open-label, phase 3 extension/switch study. The study design originally consisted of 3 periods: a titration period of 1–2 months, a stable dosing period up to 2 years, and a follow-up period of 1 week. RESTORE was approved by a central review board (WGC IRB, Princeton, NJ; reference number 20192955). The study was conducted in accordance with the ethical principles of the Declaration of Helsinki, the International Conference on Harmonization, Good Clinical Practice, and all applicable laws and regulations. Adult participants provided written informed consent, and participants aged 16 or 17 years gave assent to participate, along with written informed consent from a parent or guardian.

### 2.2 Participants

Individuals aged ≥16 years with a diagnosis of narcolepsy type 1 (NT1) or 2 (NT2) who completed the REST-ON trial, were oxybate-naive, or were on a stable dose (≥1 month) of twice-nightly IR oxybate (SXB or mixed-salt oxybates) and willing to switch to ON-SXB were enrolled in the study. Individuals taking stable doses (>3 weeks before study enrollment) of concomitant central nervous system alerting agents were included in the study. Participants were excluded if they were taking prohibited medications (ie, anticonvulsants; clonidine; hypnotics; anxiolytics; sedating antihistamines; antipsychotics; other experimental/non-experimental medications for narcolepsy, cataplexy, or any other condition; or with sedating/CNS depressant effects) or had sleep apnea with an apnea-hypopnea index >15 or requiring devices such as continuous positive airway pressure.

### 2.3 Dosing

Participants who switched from twice-nightly IR oxybate to ON-SXB (ie, switch participants) received an initial dose of ON-SXB equivalent/closest to the total nightly dose of twice-nightly IR oxybate. ON-SXB doses were adjusted in increments of 1.5 g/night weekly to a maximum dose of 9 g based on efficacy and tolerability as determined by the investigator.

### 2.4 Assessments and data analysis

Participants who switched from twice-nightly IR oxybate to ON-SXB completed a patient preference questionnaire (paper or electronic) after 3 months of stable ON-SXB treatment to evaluate their preference for a once-nightly vs a twice-nightly dosing regimen. Data from the patient preference questionnaire were analyzed using the modified intent-to-treat (mITT) population defined as participants who received ≥1 dose of ON-SXB and had ≥1 valid post-baseline efficacy assessment. At baseline, participants switching from twice-nightly IR oxybate completed a nocturnal adverse events (AE) questionnaire (paper or electronic; Table S1) to assess participant experiences with the second, middle-of-the-night IR oxybate dose in the 3 months before entering the study. Data from participants who received ≥1 dose of ON-SXB in the study (safety population) were included in the analysis of responses on the nocturnal AE questionnaire. Participants who completed the study answered an end-of-study (EOS) questionnaire to capture their experiences with ON-SXB. All data were analyzed descriptively.

## 3 Results

### 3.1 Patient disposition and demographics

The study took place in the US and Canada between March 2020 and October 2023; ClinicalTrials.gov registration was submitted on June 23, 2020, and posted on June 30, 2020. Owing to the COVID-19 pandemic, the first participant enrolled in July 2020. A total of 217 individuals were screened, 184 were enrolled in the study, and 78 (42.4 %) completed the study. The overall safety population comprised 180 participants, of whom 130 were switch participants. Results from the non-switch participants (n = 50) will be reported elsewhere. The mean (SD) age of switch participants was 36 (13.3) years. Most participants were female (71.5 %) and white (84.6 %; Table 1).

**Table 1:** Demographics of switch participants (safety population).

Characteristic	Switch Participants
	N = 130
**Mean (range) age, y**	36 (16–84)
**Median (range) BMI, kg/m**^**2**^	26.2 (16.0–45.6)
**Sex, n (%)**	
**Female**	93 (71.5)
**Male**	37 (28.5)
**Race, n (%)**	
**White**	110 (84.6)
**Black/African American**	7 (5.4)
**Asian**	6 (4.6)
**Other**	6 (4.6)
**Not Reported**	1 (0.8)

BMI, body mass index.

### 3.2 Patient preference questionnaire

A total of 98 (75.4 %) switch participants received ≥1 dose of ON-SXB, had ≥1 valid post-baseline efficacy assessment, and completed the patient preference questionnaire. Of these 98 participants, 92 (93.9 %) preferred the once-nightly dosing regimen compared to twice-nightly.

### 3.3 Nocturnal AE questionnaire

Of the 130 switch participants included in the safety population, 129 completed the nocturnal AE questionnaire at baseline. Participants were asked 18 questions (Table S1) regarding whether they had experienced, in the previous 3 months, a myriad of potential untoward events associated with waking to take the second dose.

**Fig. 1:**
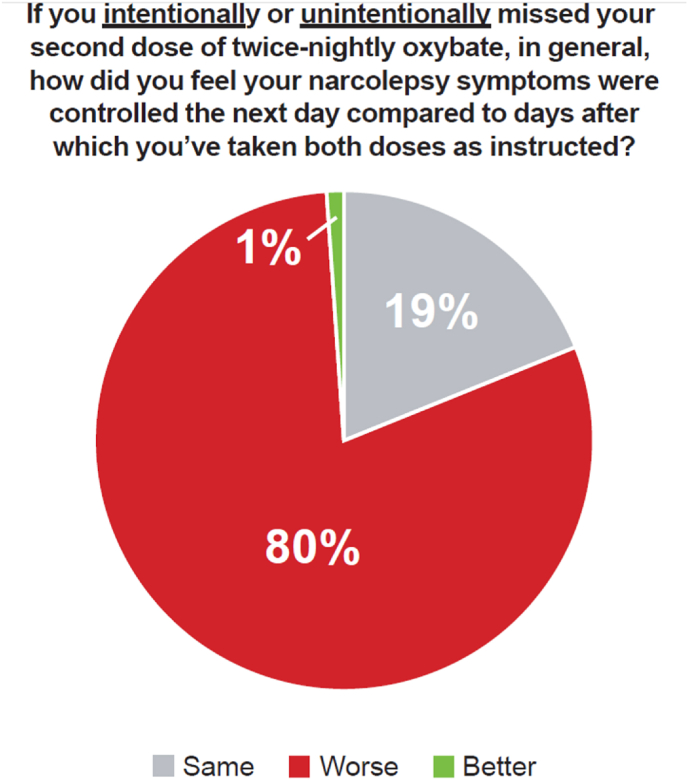
**How Participants Who Missed the Second Dose of Immediate-Release Oxybate Felt the Next Day**. Of the participants who missed their second dose (n = 110), the percentage who felt worse, same, or better the next day.

**Fig. 2:**
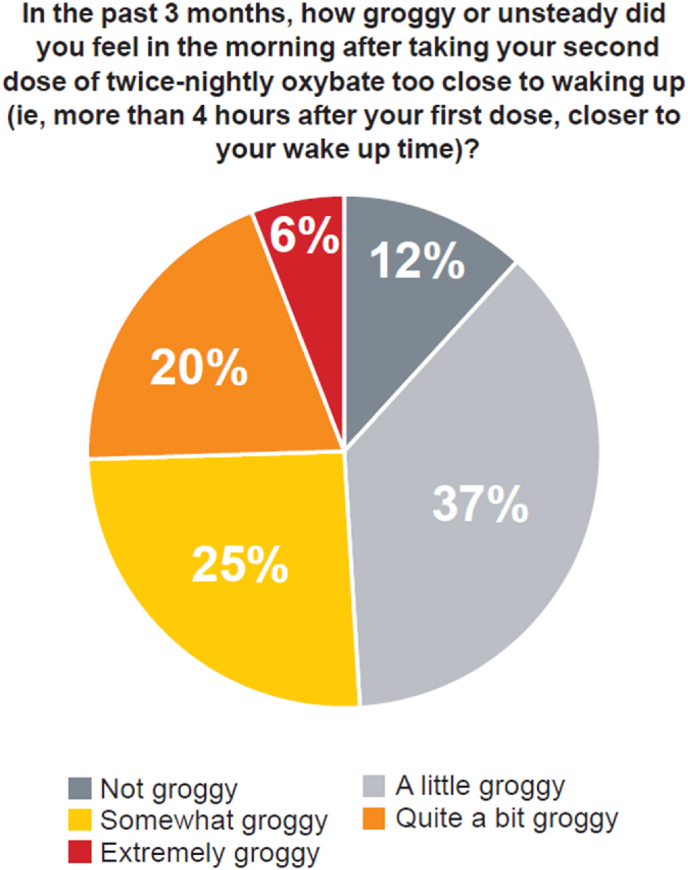
**How Participants Who Took the Second Dose of Immediate-Release Oxybate** > **4 h After the First Dose Felt the Next Day.** Of the participants who took their second dose >4 h after the first dose (n = 51), the percentage who felt “not groggy” to “extremely groggy” the next day.

Ninety (69.2 %) participants intentionally (n = 26, 20 %) and/or unintentionally (n = 84, 64.6 %) missed their second dose. When asked if their narcolepsy symptoms were better, the same, or worse the day after missing their second dose, 80 % of these participants felt that control of their symptoms was worse the next day compared to days after which they had taken both doses as prescribed (Fig. 1). Fifty-one participants (39.2 %) took their second IR oxybate dose >4 h after the first dose, with more than half feeling somewhat, quite a bit, or extremely groggy/unsteady the next day (Fig. 2).

**Fig. 3:**
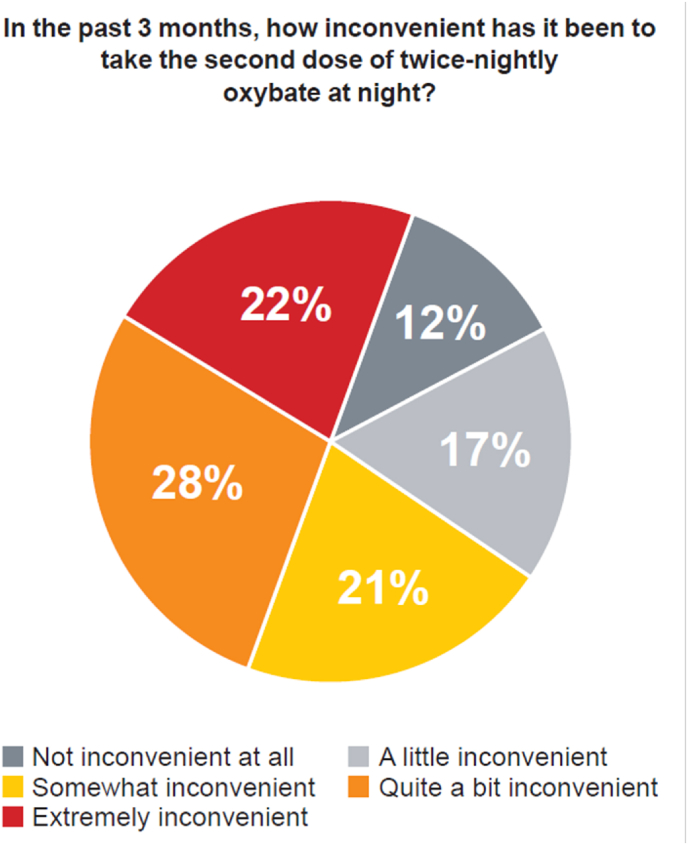
**Inconvenience of Taking the Second Dose of Immediate-Release Oxybate.** In the past 3 months, the percentage of participants (n = 130) who found taking the second dose at night inconvenient ranged from “not inconvenient at all” to “extremely inconvenient.”

A total of 120 (92.3 %; Table 2) participants reported getting out of bed after taking their second dose of oxybate in the past 3 months (eg, to go to the bathroom). Of these participants, 9 (7.5 %) reported falling after waking up for the second dose, and 5 (4.2 %) reported injuries. In the 3 months prior to baseline, 27 (20.8 %) participants reported nausea, and 38 (29.2 %) participants experienced other side effects (eg, enuresis, nocturnal eating, somnambulism) after awakening to take their second dose of oxybate. The second oxybate dose caused anxiety or other concerns for 39 participants (30 %), with 30 (23.1 %) participants requiring another person to wake up with them to ensure they took the second dose. In the past 3 months, 92 participants (70.8 %) found the second oxybate dose to be somewhat, quite a bit, or extremely inconvenient (Fig. 3).

**Table 2:** Summary of nocturnal AE questionnaire.

Question, n (%)	Switch Participants N = 130
In the past 3 months, have you ever experienced anxiety or concerns related to taking the second dose of IR oxybate?	
Yes	39 (30.0)
In the past 3 months, have you ever combined your first and second IR oxybate doses?	
Yes	4 (3.1)
In the past 3 months, have you ever taken more or less of the prescribed second dose of IR oxybate?	
More	1 (0.8)
Less	32 (24.6)
In the past 3 months, have you ever gotten out of bed after awakening to take your second dose of IR oxybate?	
Yes	120 (92.3)
If yes, have you ever fallen after awakening for your second dose of IR oxybate?	9 (7.5)^a^
If yes, have you ever injured yourself after awakening for your second dose of IR oxybate?	5 (4.2)^a^
In the past 3 months, have you experienced any other side effects (eg, bed wetting, sleep walking/eating) after awakening to take your second dose of IR oxybate?	
Yes	38 (29.2)
In the past 3 months, have you felt sick to your stomach after taking your second dose of IR oxybate?	
Yes	27 (20.8)
In the past 3 months, have you needed to do something to prevent possible negative effects of taking the second dose of IR oxybate (putting up gates/have another person wake up with you)?	
Yes	11 (8.5)
In the past 3 months, have you ever needed to have another person wake up with you to ensure you take your second dose of IR oxybate?	
Yes	30 (23.1)
In the past 3 months, has there ever been an instance when you woke up to take your second dose of IR oxybate to find that it was missing?	
Yes	1 (0.8)

a Percentage calculated with a denominator of n = 120 participants. IR oxybate includes Xyrem® (Jazz Pharmaceuticals, Inc., Palo Alto, CA) or Xywav® (Jazz Pharmaceuticals, Inc., Palo Alto, CA). IR, immediate-release.

### 3.4 EOS questionnaire

A total of 68 (52.3 %) switch participants completed the EOS questionnaire (Table 3). Since starting ON-SXB, 43/68 (63.2 %) participants rated their narcolepsy much or somewhat better. Compared to other narcolepsy treatments they had previously taken, 54/68 (79.4 %) participants were very satisfied and 11/68 (16.2 %) were somewhat satisfied with ON-SXB. When asked if they would recommend ON-SXB to a family member or friend with narcolepsy, 63/68 (92.6 %) participants stated that they would. Since beginning treatment with ON-SXB, most participants agreed that they are better able to get through the day without falling asleep (43/68; 63.2 %), sleep through the night (62/68; 91.2 %), accomplish more at work or school (40/68; 58.8 %), and socialize with friends or family (41/68; 60.3 %). In total, 62/68 (91.2 %) participants agreed that they were better able to follow the recommended medication schedule of ON-SXB than that of their previous oxybate. Additionally, 25/68 participants (36.8 %) agreed that they were able to do more daily activities since starting ON-SXB (full responses in Table S2; select illustrative examples in Table 4).

**Table 3:** Summary of end-of-study questionnaire.

Question, n (%)	Switch Participants N = 68
Since you started taking ON-SXB, how is your narcolepsy?	
Much better	23 (33.8)
Somewhat better	20 (29.4)
About the same	24 (35.3)
Worse	1 (1.5)
Compared to other treatments you've taken for narcolepsy, how satisfied are you with ON-SXB?	
Very satisfied	54 (79.4)
Somewhat satisfied	11 (16.2)
Neither satisfied nor dissatisfied	2 (2.9)
Definitely not satisfied	1 (1.5)
If your friend or a family member had narcolepsy, would you recommend ON-SXB to them?	
Yes, definitely	63 (92.6)
Not sure/no opinion	5 (7.4)
Since starting treatment with ON-SXB, would you say any of the statements below are true? If you agree, check “Yes.” If you don't agree, check “No.”	
I am better able to get through the day without falling asleep	
Yes	43 (63.2)
No	14 (20.6)
Not sure	11 (16.2)
I am better able to sleep through the night	
Yes	62 (91.2)
No	4 (5.9)
Not sure	2 (2.9)
I am getting more done at work/school	
Yes	40 (58.8)
No	11 (16.2)
Not sure	17 (25.0)
I am better able to socialize with friends or family	
Yes	41 (60.3)
No	11 (16.2)
Not sure	16 (23.5)
Since switching from IR oxybate, please tell us if you agree with the following statement. If you agree, check “Yes.” If you don't agree, check “No.”	
I am better able to follow the recommended medication schedule	
Yes	62 (91.2)
No	3 (4.4)
Not sure	3 (4.4)
Are there any daily activities that you previously could not do, or now can do better, since you started ON-SXB?	
If yes, please briefly describe	25 (36.8)
No	27 (39.7)
Not sure	16 (23.5)

ON-SXB is LUMRYZ™ (Avadel Pharmaceuticals, Chesterfield, MO). IR oxybate includes Xyrem® (Jazz Pharmaceuticals, Inc., Palo Alto, CA) or Xywav® (Jazz Pharmaceuticals, Inc., Palo Alto, CA). IR, immediate-release.

**Table 4:** Selected switch participant responses from EOS questionnaire^a^^b^

Category of Improvement	Mentions in EOS, n (%)	Illustrative Example
Improved work/school performance	7/25 (28 %)	“Succeed in career instead of just making it day-by-day with no hope for the future.”
		“Work-related travel is easier now. The TSA line is faster if I'm not bringing liquid medication, and I don't need to use alarms in the middle of the night which would bother other people sharing room.”
Improved socialization/time with family	6/25 (24 %)	“Participate w/family more, work out”
Improved sleep	3/25 (12 %)	“The biggest change for me has been in the quality of sleep. It's more like I slept before narcolepsy, I wake up gradually instead of all at once …”
Ability to perform household chores and/or exercise	6/25 (24 %)	“I can get up, get ready and run errands now. Before taking [ON-SXB] I would do everything in the afternoon after taking a nap, now I can do them in the morning.”
Better able to drive a car	8/25 (32 %)	“I am able/feel awake enough to drive more towards the end of the day/make plans and follow through with them.”

EOS, end of study; ON-SXB, once-nightly sodium oxybate.

a Select responses from switch participants who responded “yes” to the question, “Are there any daily activities that you previously could not do, or now can do better, since you started taking ON-SXB?” on the EOS questionnaire.

b If participants cited more than 1 improvement, multiple entries were accounted for.

### 3.5 Safety

Of the 130 switch participants in the safety population, 104 (80 %) participants experienced ≥1 treatment-emergent adverse event (TEAE). Most TEAEs were mild (n = 51, 49 %) or moderate (n = 40, 38.5 %) in severity. The most common TEAEs were COVID-19 (n = 21; 16.2 %), headache (n = 18; 13.8 %), nausea (n = 17; 13.1 %), nasopharyngitis (n = 13; 10 %), somnolence (n = 12; 9.2 %), enuresis (n = 11; 8.5 %), sinusitis (n = 10; 7.7 %), anxiety (n = 8; 6.2 %), dizziness (n = 7; 5.4 %), and somnambulism (n = 7; 5.4 %). Seventy-one (54.6 %) of the switch participants reported an adverse drug reaction (ADR) determined by the investigator to be related or possibly related to ON-SXB. The most common ADRs were nausea (n = 12; 9.2 %), headache (n = 10; 7.7 %), enuresis (n = 10; 7.7 %), somnolence (n = 9; 6.9 %), and falls (n = 8; 6.2 %). Serious TEAEs were reported by 8 (6.2 %) participants, although none of these were related to ON-SXB. A total of 7 (5.4 %) participants discontinued the study due to ≥1 TEAE.

## 4 Discussion

Negative patient experiences and the burden associated with taking a second, middle-of-the-night IR oxybate dose were captured in this study, as participants using twice-nightly oxybate dosing reported experiencing falls, injuries, missed second doses, late second doses, and anxiety regarding having to wake up to take an additional dose. Sleeping through the night is a primary concern for people with narcolepsy [19]. Despite the prescribing information indicating that patients should remain in bed after dosing [8,9], 92 % of switch participants reported getting out of bed after the second IR oxybate dose, with 9 reporting falls and 5 reporting injuries. Most participants (70.8 %) found taking the second dose to be extremely, quite a bit, or somewhat inconvenient. Nearly 1 out of 4 participants required assistance from someone else to wake them up in the middle of the night. Consequently, participants had an overwhelming preference (>90 %) for the once-nightly dosing regimen.

A second important finding in this study was that most participants felt their narcolepsy symptoms were improved to some degree with once-nightly dosing after switching from twice-nightly dosing (63.2 %) and had a preference for ON-SXB over other narcolepsy treatments they had taken previously (79.4 %). In addition, most participants (92.6 %) would recommend ON-SXB to a family member or friend if they had narcolepsy. Many participants reported improvements in their ability to perform daily activities (36.8 %), not fall asleep during the day, have better sleep through the night, accomplish more at work or school, and better socialize with friends or family. We speculate that the reported improvements in symptoms are related to improved adherence to the dosing schedule because the majority (91.2 %) of switch participants felt that they were better able to follow the dosing schedule of ON-SXB. These data are consistent with results of a meta-analysis of 13 studies that demonstrated significantly greater adherence and compliance rates with once-daily dosing vs dosing regimens requiring more than 1 dose per day (*P* < 0.001) [20].

ON-SXB was well tolerated, as most TEAEs were mild or moderate in severity. TEAEs were aligned with the known safety profile of IR oxybates [4,11,21]. Reported AEs were consistent with recognized oxybate AEs [8,9]. Less than 10 % of switch participants experienced a serious TEAE or discontinued RESTORE due to a TEAE.

Until ON-SXB became available in 2023, IR formulations were the only oxybate option for ≥20 years. During that time, patients taking oxybate had to manage their narcolepsy symptoms either by waking up in the middle of the night to take their second dose or to take only one dose and have exposure to therapy only during the first part of the night, an untested regimen for efficacy. The 2-dose dosing regimen has been reported to be challenging for patients. In a discrete choice experiment (DCE), patients reported that a once-nightly dosing frequency over twice-nightly was the most important factor in taking the medication as directed and reducing the patient's stress and anxiety when taking the medication [22]. An additional DCE of healthcare providers who treat patients with narcolepsy further confirmed that once-nightly over twice-nightly dosing would likely result in patients feeling less stress and anxiety when thinking of taking the medication [23]. Further, in a TREND Community survey documenting patient experience with IR oxybate, the majority of patients reported accidently missing the second dose or taking the second dose >4 h after the first dose (75 % and 59 %, respectively), which impacted their ability to function the next day, including poor sleep quality, brain fog, and grogginess [12]. Our results are in accord with these findings; within a 3 month period, approximately 70 % of participants unintentionally and/or intentionally missed their second IR oxybate dose. Doses may be unintentionally missed owing to sleeping through alarm(s) or waking >4 h after the first dose and thus being unable to take the second dose because of potential interference with next-day activities; intentionally missed doses may occur when patients need to wake earlier than their normal scheduled sleep time. Approximately 39 % of participants had taken their second dose >4 h after the first dose with resultant next-day effects of the late dosing, such as grogginess, occurring.

The TREND Community study also reported that anxiety and side effects were in the top 20 terms co-mentioned with the second oxybate dose [12]. In RESTORE, approximately 30 % of switch participants reported anxiety or concerns related to taking the second dose, 21 % of participants reported nausea, and approximately 29 % reported other side effects (eg, enuresis, nocturnal eating, somnambulism) after waking up to take their second dose.

The cumbersome middle-of-the-night dosing regimen of IR oxybates may pose other risks to patients owing to the potential for dosing errors. A study that evaluated data from FAERS found reports of safety risks occurring with the twice-nightly oxybate dosing [13]. Inappropriate dosing reports included intentionally and unintentionally taking the second IR oxybate dose <2.5 h after the first dose, taking the dose too late, or not taking the medication at all. The data from FAERS showed that accidental early dosing resulted in AEs, emergency service use, and hospitalizations [13]. A case report describing emergency care following a mistimed second dose illustrates the risk of error that can occur due to mistimed overnight redosing [14]. In this context, ON-SXB may improve patient safety as it would eliminate AEs associated with middle-of-the-night dosing errors. Further, fully adhering to the nightly dosing regimen (ie, taking a single therapeutic bedtime dose to cover a full night of sleep) likely improves treatment effectiveness and outcomes as exposure is more controlled.

This study has several limitations that merit consideration. Approximately 58 % of RESTORE participants discontinued the study early; however, it is important to recognize that RESTORE was nearly 4 years in duration. For context, discontinuation rates in open-label studies in narcolepsy have ranged from 28.7 % for a 9-month study, to 33.3 % for a 1-year study, to 33.6 % for a 6-month study [[24], [25], [26]]. Notably, only approximately 30 % of RESTORE switch participants had discontinued the study 12 months after enrollment, which is in line with these discontinuation rates from other open-label studies. RESTORE was originally designed as a 2-year open-label study, with a planned transition to commercial medication based upon the anticipated FDA approval in October 2021. However, as FDA approval was delayed owing to a patent issue [27], RESTORE was extended to approximately 3 years as a result. The protocol required participants to travel, often long distances, to their clinical site to pick up their medication each month. The COVID-19 pandemic emerged as RESTORE was initiated, and the most restricted period of travel (prior to widespread vaccine availability) persisted through the summer of 2021. One of the highest enrolling clinical sites, located in Canada, was closed by the sponsor in October 2022, as there are not currently plans to seek approval from Health Canada. Switch participants leaving the study before completing the patient preference questionnaire may have led to a bias favoring ON-SXB. The results of the nocturnal adverse event questionnaire may not be fully generalizable, as these participants opted to switch from twice-nightly dosing to once-nightly dosing; however, other data underscore the challenges with chronically waking up in the middle of the night [12,13,22,23]. Last, participants’ responses to survey questions to understand their experience with twice-nightly oxybates may have been affected by recall bias as the participants were asked to consider the previous 3 months.

## 5 Conclusion

The single bedtime dose of extended-release ON-SXB eliminates the burden associated with twice-nightly oxybate regimens and was shown to be the preferred dosing regimen in this study. Our data suggest ON-SXB reduces treatment burden of oxybate therapy in people with narcolepsy and may have an improved efficacy and safety profile considering the more streamlined and controlled exposure resulting from a single dose.

## Funding

This study was funded by Avadel Pharmaceuticals (Chesterfield, MO), which was involved in study design; collection, analysis, and interpretation of data; writing of the manuscript; and the decision to submit the article for publication.

## CRediT authorship contribution statement

**Asim Roy:** Writing – review & editing, formal analysis. **Thomas Stern:** Writing – review & editing, formal analysis. **John Harsh:** Writing – review & editing, formal analysis. **J. Douglas Hudson:** Writing – review & editing, formal analysis. **Akinyemi O. Ajayi:** Writing – review & editing, formal analysis. **Bruce C. Corser:** Writing – review & editing, formal analysis. **Emmanuel Mignot:** Writing – review & editing, formal analysis. **Adrian Santamaria:** Writing – review & editing, formal analysis. **Anne Marie Morse:** Writing – review & editing, formal analysis. **Brian Abaluck:** Writing – review & editing, formal analysis. **Sally Ibrahim:** Writing – review & editing, formal analysis. **Paula K. Schweitzer:** Writing – review & editing, formal analysis. **Katie Lancaster:** Writing – review & editing, formal analysis. **Jordan Dubow:** Writing – review & editing, formal analysis. **Jennifer Gudeman:** Writing – review & editing, formal analysis.

## Declaration of competing interest

**AR** has received grant/research support from Jazz Pharmaceuticals, Suven, Inspire, Nyxoah, LivaNova, and Avadel Pharmaceuticals; is a speaker for Avadel Pharmaceuticals; is a consultant for Jazz Pharmaceuticals, Suven, Inspire, and Avadel Pharmaceuticals; and has served on speakers bureaus for Jazz Pharmaceuticals and Eisai. **TS** is a consultant and speaker for Avadel Pharmaceuticals. **JH** has nothing to disclose. **JDH** has nothing to disclose. **AOA** is a consultant and speaker for Avadel Pharmaceuticals. **BCC** is a member of the speakers bureaus of Jazz Pharmaceuticals; Merck & Co., Inc.; Eisai; and Harmony Biosciences. He is an advisor for and has received consulting fees and honoraria from Avadel Pharmaceuticals. **EM** has received clinical trial or research funding from Apple, Avadel Pharmaceuticals, Eisai, Jazz Pharmaceuticals, Suven, and Takeda; owns stocks in Centessa and Sleep Apnea Co; and has consulted for or received honorarium from Avadel Pharmaceuticals, Idorsia, Jazz Pharmaceuticals, and Takeda. **AS** has received research grants from Avadel Pharmaceuticals and Harmony Biosciences and has served on speakers bureaus for Avadel Pharmaceuticals, Jazz Pharmaceuticals, and Axsome Pharmaceuticals. **AMM** has served as a consultant, speaker, and/or on advisory boards for Avadel Pharmaceuticals, Eisai, Harmony Biosciences, Jazz Pharmaceuticals, NLS Pharmaceuticals, Alkermes, and Takeda Pharmaceutical Co; has received grant funding from National Institutes of Health, UCB Pharmaceuticals, Jazz Pharmaceuticals, ResMed Foundation, Coverys Foundation, and Geisinger Health Plan; is the CEO of DAMM Good Sleep, LLC; and has served as an advisor for Neura Health. **BA** is an employee of Avadel Pharmaceuticals. **SI** is affiliated with a hospital that received funding for the RESTORE trial and has received clinical trial research funding from Jazz Pharmaceuticals, Harmony Biosciences, and the National Institutes of Health. **PKS** received payment from Apnimed Inc., as a consultant. Her institution has received research funding from Apnimed Inc., Avadel, Harmony Biosciences, Inspire Medical, and Jazz Pharmaceuticals. **KL** was enrolled as a study participant in the RESTORE study. Following her participation in RESTORE, KL has received compensation from Avadel for speaking activities related to sharing her experience as a person with narcolepsy and with ON-SXB. **JD** was an employee of Avadel Pharmaceuticals at the time of the trial. **JG** is an employee of Avadel Pharmaceuticals.
