# Supplementary material for: RESTORE: Once-nightly oxybate dosing preference and nocturnal experience with twice-nightly oxybates
Source: Sleep Med X. 2024 Aug 15;8:100122. doi: 10.1016/j.sleepx.2024.100122 (PMC11388664; doi:10.1016/j.sleepx.2024.100122)
Supplement: Multimedia component 1 [file mmc1.docx]

# Supplementary

**Supplementary Table S1. Nocturnal Adverse Event Questionnaire**. The questionnaire was given at baseline to participants who switched from a stable dose of twice-nightly immediate-release (IR) oxybate to once-nightly sodium oxybate. This questionnaire evaluated nocturnal adverse events experienced in the last 3 months when on twice-nightly IR oxybate before entering the study.

| **Question** | **Check Answer** |
| --- | --- |
| In the past 3 months, was there a night where you took your first dose of IR oxybate, but **intentionally** missed your second dose (in other words, you avoided taking the  second dose on purpose)? | No  Yes |
| In the past 3 months, was there a night where you took your first dose of IR oxybate, but **unintentionally** missed your second dose (for example, you slept through or  forgot to set your alarm)? | No  Yes |
| As a follow up to question 1 or 2, if you intentionally or unintentionally missed your second dose of IR oxybate, in general, how did you feel your narcolepsy symptoms were controlled the next day compared to days after which you’ve taken both doses as instructed? | Better  The same  Worse |
| In the past 3 months, have you ever experienced anxiety or concerns related to taking the second dose of IR oxybate? | No  Yes |
| In the past 3 months, have you ever **combined your first and second IR oxybate** **doses** (i.e., taken them at the same time)? | No  Yes |
| In the past 3 months, have you ever taken **more** IR oxybate than prescribed when you were taking your second dose? | No  Yes |
| In the past 3 months, have you ever taken **less IR oxybate** than prescribed when you were taking your second dose? | No  Yes |
| In the past 3 months, have you ever taken your second dose of IR oxybate **after it was scheduled** (more than 4 hours after the first dose)? | No  Yes |
| In the past 3 months, how **groggy or unsteady** did you feel in the morning after taking your second dose of IR oxybate too close to waking up (i.e. more than 4 hours after your first dose, closer to your wake up time)? | Not groggy or unsteady at all  A little groggy or unsteady Somewhat groggy or unsteady  Quite a bit groggy or unsteady  Extremely groggy or unsteady |
| In the past 3 months, how **inconvenient** has it been to take the second dose of IR oxybate at night? | Not inconvenient at all  A little inconvenient  Somewhat inconvenient  Quite a bit inconvenient  Extremely inconvenient |
| In the past 3 months, have you ever **gotten out of bed** after awaking to take your second dose of IR oxybate (for example, to go to the bathroom)? | No  Yes |
| If yes, in the past 3 months, have you ever **fallen** after awaking to take your second dose of IR oxybate? | No  Yes |
| If yes, in the past 3 months, have you ever **injured yourself** after awaking to take your second dose of IR oxybate? | No  Yes |
| In the past 3 months, have you ever experienced any other side effects (for example, bed wetting, nocturnal eating, sleep walking, etc.) after waking up to take your second dose of IR oxybate? | No  Yes |
| In the past 3 months, have you ever felt **sick to your stomach** after taking your second dose of IR oxybate? | No  Yes |
| In the past 3 months, have you ever needed to do anything to prevent possible negative effects of **taking the second dose of IR oxybate** (for example, putting up gates to prevent sleepwalking, having another person wake up with you)? | No  Yes |
| In the past 3 months, have you ever needed to have another person wake up with you to ensure you take your second dose of IR oxybate? | No  Yes |
| In the past 3 months, has there ever been an instance when you woke up to take your second dose of IR oxybate, but it was missing (for example, taken by another person, spilled by pet)? | No  Yes |

IR oxybate includes Xyrem^®^ (Jazz Pharmaceuticals, Inc., Palo Alto, CA) or Xywav^®^ (Jazz Pharmaceuticals, Inc., Palo Alto, CA). IR, immediate-release.

Supplementary Table S2. End-of-Study Survey Open-Ended Responses. An end-of-study survey was administered to switch participants who completed the study. Switch participants who answered yes (n=25) to the final question of the survey provided an open-ended response describing the daily activities they are now able to complete since starting ON-SXB. IR, immediate-release; ON-SXB, once-nightly sodium oxybate.

| Are there any daily activities that you previously could not do, or now can do better, since you started ON-SXB? If yes, please briefly describe. |
| --- |
| Working out |
| Better alertness and functioning at work. Better cognitive system engineering |
| Succeed in career instead of just making it day-by-day with no hope for the future |
| He stated he can now work a consistent job and can take college courses online. His least wakeful hours 3 pm to night are nowhere as bad as they were when he didn't have [IR oxybate] or [ON-SXB] product |
| Just waking up/keeping a sleep schedule and being able to consistently keep a job and go to school which I am incapable of when not medicated |
| Stay awake during the day |
| Driving and being able to somewhat keep up with socializing |
| Stay up longer in the day and concentrate more at school |
| The biggest change for me has been in the quality of my sleep it's more like I slept before narcolepsy, I wake up gradually instead of all at once and the length of time that I can expect to sleep is... |
| Daily routines in the morning are easier, driving much longer distances |
| I can control my emotions much better. I can juggle multiple tasks better. My stimulant is more effective |
| Participate w/family more, work out |
| Watch movies in theatre, stay awake in a car |
| Drive longer and enjoy more hobbies |
| I am able/feel awake enough to drive more towards the end of the day/make plans and follow through with them |
| Driving is easier |
| I am able to drive a little further without sleep attacks |
| Travel is easier since its powder versus liquid. Only need to take 1 dose means my sleep schedule is more consistent |
| Work-related travel is easier now. The TSA line is faster if I’m not bringing liquid medication, and I don't need to use alarms in the middle of the night which would bother other people sharing room |
| Subject can drive at night, sit and talk after a meal, sit still and not be as distracted, finds it easier to process conversations |
| The participant feels she can cook more or better without being exhausted. More focused and can do household chores better that earlier, feels active and can concentrate better on errands |
| I can get up, get ready and run errands now. Before taking [ON-SXB] I would do everything in the afternoon after taking a nap, no I can do them in the morning |
| Household chores, walking the dog, attending regularly scheduled events |
| Socializing; working out; work |
| Socialization with friends & classmates without feeling depleted, drive more, endure long days |

ON-SXB is LUMRYZ™ (Avadel Pharmaceuticals, Chesterfield, MO). IR oxybate includes Xyrem® (Jazz Pharmaceuticals, Inc., Palo Alto, CA) or Xywav® (Jazz Pharmaceuticals, Inc., Palo Alto, CA).
